# Plasmacytoid dendritic cells are increased in cerebrospinal fluid of untreated patients during multiple sclerosis relapse

**DOI:** 10.1186/1742-2094-8-2

**Published:** 2011-01-07

**Authors:** Ana Leda F Longhini, Felipe von Glehn, Carlos Otávio Brandão, Rosemeire FO de Paula, Fernando Pradella, Adriel S Moraes, Alessandro S Farias, Elaine C Oliveira, Juan G Quispe-Cabanillas, Cassiana Horta Abreu, Alfredo Damasceno, Benito P Damasceno, Konstantin E Balashov, Leonilda MB Santos

**Affiliations:** 1 Neuroimmunology Unit, Dept Genetics, Evolution and Bioagents, Biology Institute University of Campinas - UNICAMP; 2 Dept of Neurology, University of Campinas - UNICAMP, Campinas, SP, Brazil; 3 FATEC-Sorocaba, SP, Brazil; 4 Robert Wood Johnson Medical School, New Brunswick, NJ, USA

## Abstract

The plasmacytoid dendritic cells (pDCs) express a high level of Toll-like receptor 9 (TLR-9), which recognizes viral DNA. Activated via TLR-9, pDCs also secrete large amounts of type I interferon which are involved either in stimulation or down regulation of immune response in multiple sclerosis (MS). In the present study, we determinate pDCs levels by flow cytometry in Cerebrospinal Fluid (CSF) and Peripheral Blood from MS patients in relapsing and in remitting phases of the disease, comparing with other non-inflammatory diseases (OND). We provide evidence that MS patients in relapse without any treatment have a significantly (p < 0.01) higher percentage of pDCs in CSF than do patients in remission or those with OND. No change in the percentage of pDCs was observed in the peripheral blood of any of these patients. The increase of pDCs in central nervous system during relapse may be explained either by a virus infection or a down regulatory process.

## Introduction

The pathogenesis of multiple sclerosis (MS) is mainly driven by central nervous system-invading encephalitogenic CD4 T lymphocytes of both the Th1 and Th17 types. These effector cells can be down-regulated by regulatory T lymphocytes [1]. One subset of dendritic cells, the plasmacytoid dendritic cells (pDCs), has been given particular emphasis due to its importance in stimulating or down regulating effectors T cells in MS [2].

These pDCs are present in the cerebrospinal fluid (CSF), leptomeninges and demyelinating lesions of patients with MS [3]. These cells express a high level of Toll-like receptor 9 (TLR-9), which recognizes viral DNA. Activated via TLR-9, pDCs secrete large amounts of type I interferon [4]. The use of type I interferon as an immunomodulator in the treatment of MS patients has proved beneficial for patients with the relapsing/remitting form of MS (RRMS), and the production of this cytokine by the pDCs may suggest an important immunomodulatory function of these cells.

In the present study, the concentration of pDCs in the CSF and peripheral blood of MS patients during relapsing and remitting phases of the disease was determined and compared to what is present in other non-inflammatory neurological diseases (OND).

## Patients and Methods

Peripheral venous blood (5 ml) and CSF (5-10 ml) samples were collected from patients with RRMS, as defined by the revised McDonald criteria [5]. The MS patients were divided into two groups: relapsing (six patients) and in remission (eleven patients). Moreover, samples were collected from 8 patients with other non-inflammatory neurological diseases (OND). Relapse was defined as recent onset (within 1-7 days) of clinical neurological symptoms, but without any clinical or laboratorial signs of infection at the time of lumbar puncture. All patients included agreed to participate in the study, which was approved by the University of Campinas Committee for Ethical Research, and they signed a term of Consent. The clinical characteristics of the patients are presented in Table 1.

**Table 1:** Demographic and baseline clinical characteristics of patients and controls

	Patients #	Age (years*)	Gender F/M	Time from first relapse (Years*)	CSF cells/μl *	Oligoclonal Bands
**RRMS - Relapse**	6	34 (30-47)	4/2	5 (1-8)	6 (0-17)	6+/0-

**RRMS- remission**	11	34 (26-61)	9/2	3 (1-8)	3 (1-23)	8+/3-

OND**	8	46 (30-64)	7/1	-	2 (0-5)	

*Median (range)

**Other Neurological Diseases

Patients using corticosteroids or other immunosuppressive and immunomodulatory drugs at the time of investigation were excluded from the study. The group with OND consisted of eight patients with no clinical evidence of any inflammatory process in the central nervous system (CNS). Two patients had had an ischemic stroke, two patients had pseudotumor cerebri, one had psychiatric disorders, one had epilepsy, one had normal pressure hydrocephalus and one patient had post trauma headache.

### Flow Cytometry Analysis

The proportion of pDCs (in %) in relation to other mononuclear cells was determined by staining the CSF and peripheral blood mononuclear cells (PBMC) with anti-human BDCA2-mAb conjugated with APC (Miltenyi Biotec, Germany). Data were acquired for gating mononuclear cells using a BD FACSCanto cytometer (BD Biosciences, USA) and analyzed using BD FACSDiva software (BD Biosciences, USA). The *p *value was determined using unpaired T-test.

## Results and Discussion

**Figure 1:**
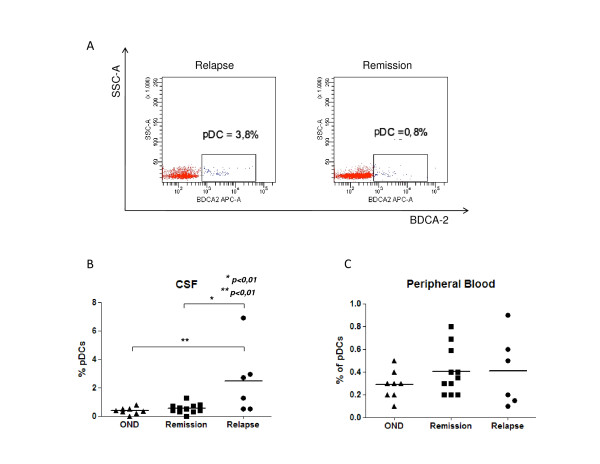
**Concentration of pDCs in relation to other mononuclear cells in CSF and peripheral blood**. **(A) **BDCA2 positive gated CSF cells, dot plot representative of MS patients in relapse and in remission. **(B) **Percentage of pDCs in CSF, with data points representing individuals of MS patients in relapse (6) and in remission (11) and in other neurological diseases (8). **(C) **Concentration of pDCs in peripheral blood, with data points representing individuals. Data are shown as median and the statistically significant differences are indicated by *(p < 0.01).

The number of pDCs is significantly elevated in the CSF of patients in the relapse phase of untreated MS compared to patients in remission (Figure 1). Since there are no differences in the number of these cells neither in the PBMC nor in total number of cells in the CSF of the same patients, pDCs must selectively increase in the CNS during the relapse phase of disease. As far as we know, this is the first observation of such an increase in the percentage of pDCs in the CSF of MS patients in a specific phase of the disease. A previous study reported an elevated concentration of dendritic cells, mainly pDCs, in patients with infections and other inflammatory neurological diseases, including MS, but no mention was made of variations during different phases of the disease [6].

The ambivalent function of pDCs has been observed in experimental autoimmune encephalomyelitis (EAE), a model for studying MS. A recent report shows that they promote priming of autoimmune Th17 in EAE, whereas depletion of pDC prior to induction of the disease decreases its severity [7]. Another recent study has demonstrated that clinical signs of EAE are exacerbated considerably if the pDCs are depleted during the peak period of the disease [2]. Thus, pDC depletion significantly enhances the activation of CNS cells and the production of cytokines such as IL-17 and IFN-γ, but not peripheral CD4 T cells [2]. Recent study developed in the EAE model, demonstrated that the tolerogenic property of pDCs is associated with MHC class II molecule in the presenting of neuroantigen to CD4 T cells. This specific-antigen stimulation induces regulatory T cells, which results in the reduction of the disease severity [8]. Moreover, pDCs also produce indolamine 2, 3 dioxigenase (IDO), which is an enzyme activated by both type I and type II Interferon, and is involved in tryptophan catabolism. Its immunosuppressive effect is linked to the reduction of local tryptophan concentration and to the activation of regulatory T cells [9,10].

Also of relevance is the impact of treatment with immunomodulators and the length of its use on the presence of pDCs in CSF. There is no significant difference in the percentage of pDCs in the CSF when we analyzed the group of MS patients treated and not treated with interferon beta IFNβ (data not shown), suggesting that the increase of pDCs is restricted to patients in relapse.

Although the exact function of pDCs in the CNS needs to be elucidated in future studies, the presence of pDCs during the phase of relapse may be explained either by a virus infection or by the regulation of inflammatory process. As the immune response evolves, the increase in the production of pro-inflammatory cytokines such as IFNs stimulates the secretion of IDO by pDCs, which in turn will activate regulatory T lymphocytes. This immunomodulatory response will probably contribute to the reduction of inflammation in the CNS, thus preparing this microenvironment for the remission phase of MS.

## Competing interests

The authors declare that they have not competing interests. KEB has served as consultants for Biogen, TEVA Neuroscience, Bayer Healthcare, and EMD Serono.

## Authors' contributions

LMBS designed the study. FVG and COB selected the patients and collected the CSF and peripheral blood. ALFL, FVG, FP, ASM, RFOP, JGQC, ECO and ASF performed the experiments. ALFL made the flow cytometry analysis. ALFL, ASF, FVG, LMBS and COB analyzed the results. ALFL, FVG, COB, ASF, CHA, AD, BPD, KEB and LMBS helped write the paper. All authors have read and approved the final version of the manuscript.
